# Peripheral immune cell traits and Parkinson’s disease: A Mendelian randomization study

**DOI:** 10.1371/journal.pone.0299026

**Published:** 2024-03-05

**Authors:** Jie Tian, Chunyan Zuo, Jingjing Shi, Dongrui Ma, Changhe Shi

**Affiliations:** 1 Zheng Zhou Railway Vocational and Technical College, Zhengzhou, Henan, China; 2 Department of Neurology, The First Affiliated Hospital of Zhengzhou University, Zhengzhou University, Zhengzhou, Henan, China; University College London, UNITED KINGDOM

## Abstract

**Background:**

The peripheral immune system is altered in Parkinson’s disease (PD), but the causal relationship between the two remains controversial. In this study, we aimed to estimate the causal relationship between peripheral immune features and PD using a two-sample Mendelian randomization (MR) approach.

**Methods:**

Genome-wide association study (GWAS) data of peripheral blood immune signatures from European populations were used for exposure and PD summary statistics were used as results. We conducted a two-sample MR study using the inverse-variance weighted (IVW), MR-Egger, and weighted median methods to evaluate the causal association between these factors. MR-Egger and MR-PRESSO were used for sensitivity analysis to test and correct horizontal pleiotropy.

**Results:**

A total of 731 immune traits were analyzed for association with PD using three MR methods. After adjustment for FDR, we observed four peripheral immunological features associated with PD using the IVW method, including expression of *CX3CR1* on monocytes [OR: 0.85, 95% CI: (0.81, 0.91), P = 6.56E-07] and *CX3CR1* on CD14+CD16+ monocytes [OR: 0.87, 95% CI: (0.82, 0.93), P = 9.95E-06].

**Conclusions:**

Our study further revealed the important role of monocytes in PD and indicated that *CX3CR1* expression on monocytes is associated with a reduced risk of PD.

## Introduction

Parkinson’s disease (PD) is the second most prevalent neurodegenerative disorder, characterized by bradykinesia, tremors, and postural instability [1,2]. The major cause of PD is the degeneration of dopaminergic neurons in the substantia nigra [3,4]. It has been observed that the immune system contributes to the progression of PD [5,6]. CNS inflammation, including reactive microglia and astrocyte activation, is heightened in PD patients [7–9]. However, the relationship between the central immune system and the peripheral immune system in PD remains unclear. Studies have indicated that peripheral immune cells migrate to the CNS and trigger inflammation. Additionally, circulating immune cells, immune proteins, and cytokines have all been linked to PD [10].

To explore the link between peripheral immune properties and PD, we employed the Mendelian randomization (MR) approach [11]. MR assesses the causal relationship between exposure and an outcome and is utilized to investigate disease risk factors [12]. Using data from a large-scale European genome-wide association study (GWAS) of peripheral blood immune signatures, we conducted a two-sample MR analysis and found that the expression of *CX3CR1* in monocytes was associated with the risk of PD.

## Materials and methods

### Exposure

We obtained genetic data for 731 immune factors (listed in S1 Table) assessed in a general population cohort of 3,757 individuals who are native to the central east coast of Sardinia, Italy. These factors include 118 absolute cell counts, 389 median fluorescence intensities of surface antigens, 32 morphological parameters, and 192 relative counts, totalling approximately 22 million genetic variants [13]. To apply the MR method, the instrumental variables (IV) were selected based on the following three assumptions: I) the variables used as instruments are related to the risk exposure of interest; (II) The variable should not be associated with any confounders between exposure and outcome; III) Variables are associated with outcomes only through their effect on exposure.

### Outcomes

For our study, we utilized PD GWAS data from datasets publicly available on the International Parkinson’s Disease Genomics Consortium (IPDGC) as of 2019, excluding 23andMe data [14]. The PD GWAS dataset comprised 33,674 PD cases and 449,056 controls, including data from the IPDGC’s NeuroX (5,851 cases, 5,866 controls), the System Genomics of Parkinson’s Disease (SGPD) Consortium (1,169 cases, 968 controls), and additional IPDGC data (8,036 cases, 5,863 controls). A geographical comparison of our samples revealed no overlap with the exposures outlined in the GWAS. Details on the samples are available in the respective studies’ supplementary materials. The subjects in both the exposure and outcome datasets included in this study were of European ancestry. No ethical approval was required, as our study was a secondary analysis of previously published data.

### Instrument selection

To identify significant SNPs for exposures, we first selected those with genome-wide significance (P < 1.00e^−6^) and a minor allele frequency (MAF) greater than 0.01 [15,16]. Second, recognizing that many SNPs in a GWAS may be in close linkage disequilibrium, we carried out a clumping procedure. This process, using European reference samples from the 1000 Genomes Project, involved setting an R^2^ threshold of less than 0.001 and a window size of 10,000 kb, retaining only the SNP with the lowest P-value. Third, we extracted SNPs related to exposure from the outcome GWAS summary data. In cases where an exposure SNP wasn’t available in the outcome GWAS, we substituted it with a proxy SNP that had a linkage disequilibrium with the exposure SNP (minimum LD r-squared value of 0.8) [17]. The fourth step involved harmonizing the exposure and outcome SNPs. During this stage, we eliminated ambiguous SNPs where the effect allele could not be clearly determined and conducted a thorough review of palindromic SNPs in the original datasets to prevent inadvertent reverse effects. The effectiveness of the genetic instrument was assessed using F-statistics, discarding any weak instrument with an F-statistic lower than 10. The F statistic is calculated as F = F = R^2^ (N−2)/(1−R^2^), where R^2^ is the variance in exposure explained by the instrumental SNPs and N is the sample size. SNPs selected through these stringent criteria were then utilized as IVs in our subsequent two-sample MR analysis.

### Two-sample Mendelian randomization analysis

The two-sample MR analysis was conducted using the Two-Sample MR package (version 0.5.6) in R software (version 4.2.1). Using the inverse variance weighted (IVW) method as the primary statistical model, this method produces the most reliable causal estimates, but it is relatively susceptible to pleiotropy and outliers [18–22]. Therefore, the Weighted Median method and MR-Egger method were employed in the following sensitivity analyses to evaluate the robustness of associations and potential pleiotropy. The weighted median method can provide a robustness check for the IVW method, being more robust to certain invalid iIVs [23]. MR-Egger regression can detect and adjust for the direct effects of the IVs on the outcome (known as horizontal pleiotropy), but its statistical power is usually lower than that of the IVW method [12].

### Sensitivity analysis

Cochran’s Q statistics were conducted to test IVs heterogeneity, with no heterogeneity determined when P > 0.05. MR-Egger intercept and MR-PRESSO global test were used to examine the existence of pleiotropy. MR-PRESSO global test was employed using the MR-PRESSO package (version 1.0). When horizontal pleiotropy exists, the MR-PRESSO outlier test was used to correct it [24]. Leave-one-out (LOO) analysis was used to evaluate if the results were driven by a single SNP. Since the outcome is a binary variable, an odd ratio (OR) was used to represent causality. Statistical power analysis for exposure was performed using the publicly available tool mRnd [25] (https://shiny.cnsgenomics.com/mRnd/). The type I error rate was set at 0.05. We used the Benjamini–Hochberg method that controls the false discovery rate (FDR) for multiple testing [26]. The code for reproducing the above analysis can be found in S1 File.

### Ethics statement

This study is entirely predicated on publicly available GWAS data, which had previously undergone and secured the necessary ethical approvals upon its publication.

## Results

After the selection and harmonization of IVs, we utilized 4,423 SNPs for MR analysis. All SNPs had F statistics above 10, demonstrating their suitability as strong instruments. The harmonized data are presented in the S2 Table.

**Fig 1:**
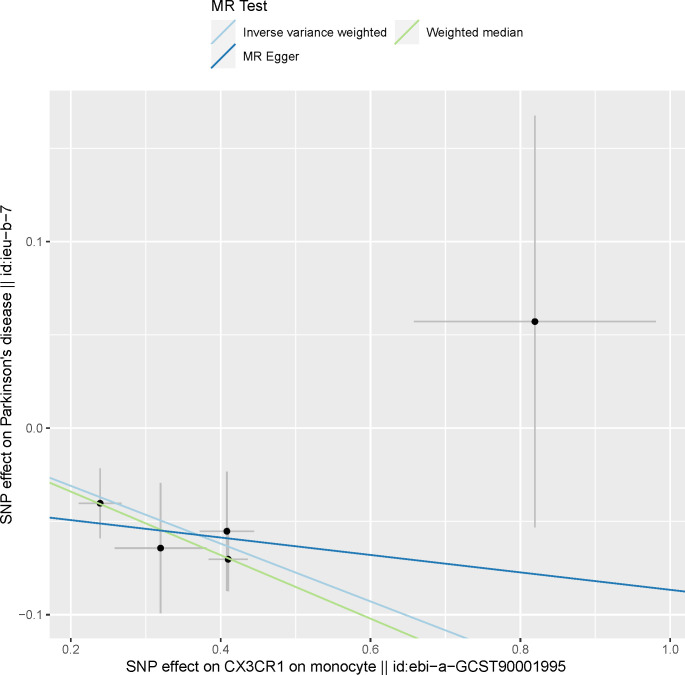
Scatter plot showing the causal effect of *CX3CR1* on monocytes on Parkinson’s disease. MR, Mendelian Randomization.

**Fig 2:**
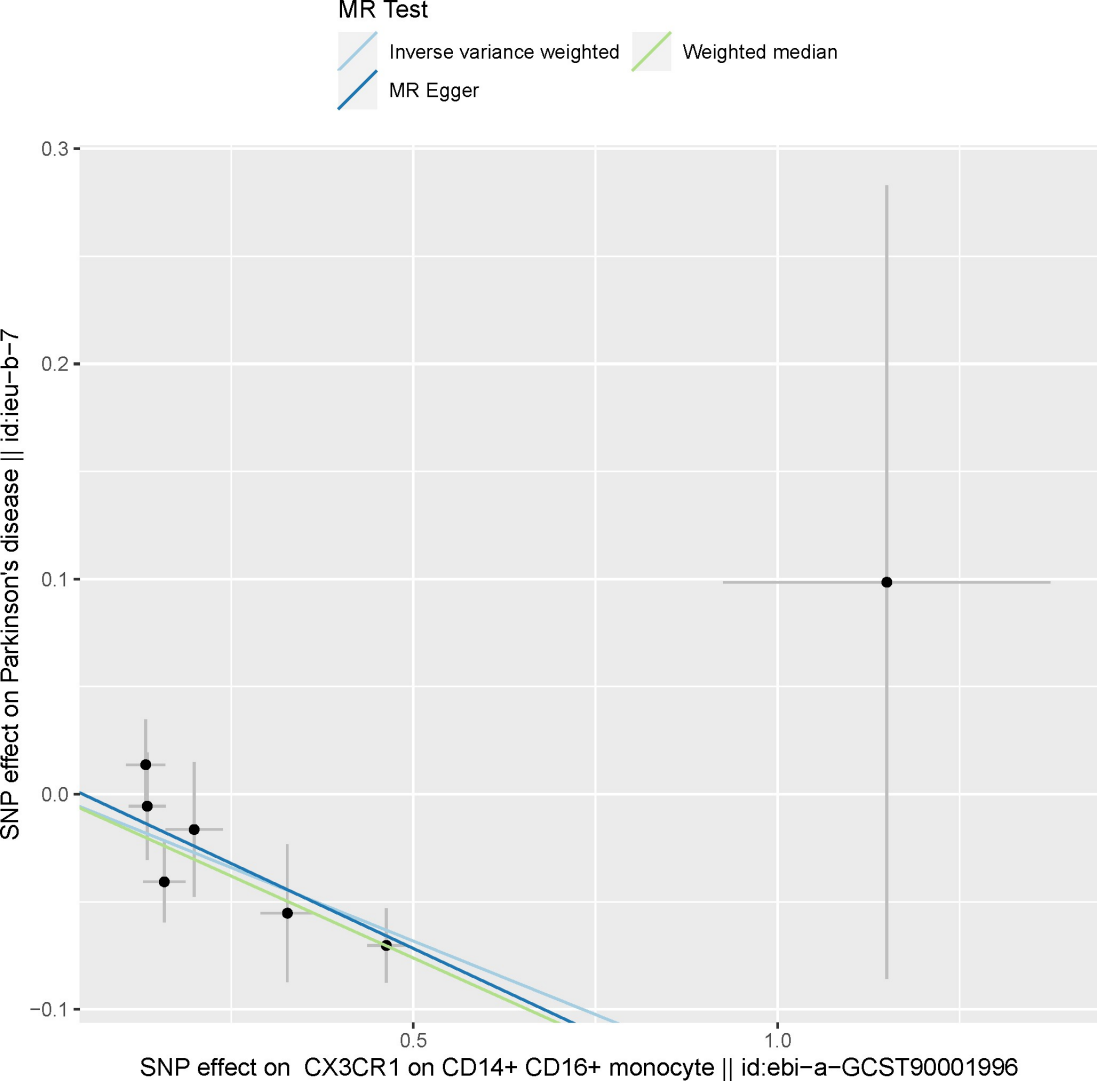
Scatter plot showing the causal effect of *CX3CR1* on CD14+ CD16+ monocyte on Parkinson’s disease. MR, Mendelian Randomization.

A total of 731 immune traits were analyzed for their association with PD using three MR methods. The results of the MR analysis of these immune cell traits are shown in Tables 1 and S3–S6. Our study findings, demonstrated through IVW analysis, reveal a strong causal relationship between increased susceptibility to PD and a reduction in *CX3CR1* levels in monocytes and CD14+ CD16+ monocytes. These estimates include a reduction in *CX3CR1* on monocytes [OR: 0.85, 95% CI: (0.81, 0.91), P = 6.56E-07] and *CX3CR1* on CD14+CD16+ monocytes [OR: 0.87, 95% CI: (0.82, 0.93), P = 9.95E-06], as detailed in Figs 1 and 2 and S3 Table. These findings remain significant even after adjusting for FDR, as shown in Table 1. The results of MR Egger and weighted median methods tend to be in the same direction.

**Table 1:** Summary of the causal relationships of 2 immune cell traits on Parkinson’s disease with various Mendelian randomization methods.

Exposure	SNP N	IVW		Weighted median		MR-Egger	
		OR (95%CI)	P_fdr_	OR (95%CI)	P_fdr_	OR (95%CI)	P_fdr_
*CX3CR1* on monocyte	5	0.85(0.81, 0.91)	4.37E-04	0.84(0.78,0.91)	2.66E-03	0.95(0.75,1.21)	0.99
*CX3CR1* on CD14+ CD16+ monocyte	7	0.87(0.82, 0.93)	3.31E-03	0.86(0.80,0.92)	3.47E-03	0.91(0.77,1.07)	0.99

Abbreviations: PD, Parkinson’s disease; MR, Mendelian randomization; IVW, inverse variance weighted; SNP, single nucleotide polymorphism; OR, odds ratio; CI, confidence interval.

We also conducted a sensitivity analysis of the MR results as shown in Table 2. In Cochran’s Q test, we observed no heterogeneity among SNPs exposed to a certain exposure. Heterogeneity analysis was consistent with other exposures, and no significant differences were found. MR-PRESSO Global test and MR-Egger test indicated no notable horizontal or directional pleiotropy across SNPs in the causal estimates relating the levels of *CX3CR1* on monocytes and CD14+ CD16+ monocytes to PD. LOO analysis further revealed that no SNP introduced pleiotropic bias in our causal estimates (S1 and S2 Figs). Both MR analysis exhibit relatively high power (Exposure: *CX3CR1* on monocyte, power: 1.00; Exposure: CX3CR1 on CD14+ CD16+ monocyte, power: 1.00). In addition, we performed pleiotropy analyses using the PhenoScanner database and did not find any other relevant characteristics of instrumental SNPs affecting PD.

**Table 2:** Results of sensitivity analysis for traits that showed significant correlations with Parkinson’s disease.

Exposure	Cochran’s Q test		MR-PRESSO			MR-Egger	
	IVW	MR-Egger	Global test P	corrected P	Distortion P	Intercept	P-value
CX3CR1 on monocyte	0.52	0.50	0.632	NA	NA	-0.04	0.42
CX3CR1 on CD14+ CD16+ monocyte	0.44	0.33	0.546	NA	NA	7.31E-03	0.72

## Discussion

Based on a large amount of publicly available genetic data, we explored the causal relationship between 731 immune traits and PD. To our knowledge, this is the first MR analysis to investigate the causal links between multiple immune phenotypes and PD. In our study, we identified significant causal relationships between PD and two immune phenotypes out of four immune traits (MFI, RC, AC, and MP), with an FDR less than 0.05. Our findings suggest that the expression of *CX3CR1* on monocytes is associated with a reduced risk of PD.

Previous research has indicated a close link between PD and alterations in peripheral immunity [27,28]. While granulocytosis, lymphocytopenia, and monocytopenia have been observed in PD patients [29], recent evidence, suggests that PD patients may exhibit normal monocyte counts [10,30]. However, alterations in monocyte populations and functions, including an increased proportion of classical CD14+CD16- monocytes and a decreased proportion of nonclassical CD14-CD16+ monocytes, have been noted [31,32]. These changes in monocyte subtypes and functions may contribute to CNS inflammation and the pathogenesis of PD, but the precise relationship between peripheral immune cell phenotype and PD requires further exploration [10]. This study was conducted using a two-sample MR analysis based on large GWAS cohorts with over 450,000 individuals, ensuring high statistical power. Our conclusions are based on genetic IVs and were inferred using various MR methods, demonstrating robustness and resilience to pleiotropy and other potential confounders.

*CX3CR1* is the receptor for *CX3CL1*, a chemokine belonging to the *CX3C* family. In the central nervous system, neurons abundantly express *CX3CL1*, while CX3CR1 is exclusively present in microglia. This *CX3CL1-CX3CR1* signalling forms the most critical communication pathway between neurons and microglia [33]. The expression of *CX3CL1* in neurons and its receptor *CX3CR1* in microglia jointly facilitate the maturation and functional regulation of these cell types, playing a vital role in coordinating various aspects of brain function [33]. The interaction between *CX3CL1* and *CX3CR1* not only promotes precise communication between microglia and neurons but also plays a significant role in neuroprotection and anti-inflammatory responses [34], involving the establishment of neural networks, modulation of synaptic maturation and plasticity, regulation of cognitive functions, and control of immune processes [35].

Previous research has demonstrated that high levels of endogenous *CX3CL1* expressed in neurons restrict the activation of *CX3CR1*, thereby maintaining microglial quiescence and inhibiting neuroinflammation [36]. Conversely, down-regulation of the *CX3CL1/CX3CR1* pathway can promote microglial activation and stimulate the release of inflammatory cytokines. Recent studies also have revealed that the chemokine *CX3CL1* and its receptor *CX3CR1* play a significant role in regulating the inflammatory response in PD [37]. When the neurotoxins *MPTP* or *6-OHDA* were administered to mice lacking the microglial receptor *CX3CR1*, the loss of dopaminergic neurons was observed to be more pronounced. In contrast, up-regulation of the *CX3CL1/CX3CR1* pathway exhibits a neuroprotective effect in the PD model, as exogenous *CX3CL1/CX3CR1* counters neuronal cell death in the striatum and leads to a significant decrease in microglial cells. Similarly, up-regulation of *CX3CL1/CX3CR1* expression in the α-synuclein PD model demonstrates a neuroprotective effect [33,38–40].

*CX3CR1* expression is also important for monocyte function, and our study found that enhanced function of resident monocyte subsets may have a protective effect on PD. Existing studies suggest that monocytes play a crucial role in PD, with alpha-synuclein inducing monocyte activation and peripheral monocytes invading the CNS in animal models of PD and other neurodegenerative diseases [38,40]. Additionally, *CX3CR1* may have a protective effect on alpha-synuclein-induced neurotoxicity, while the mechanism by which *CX3CR1* expression reduces the risk of PD remains unclear and requires further investigation [33,36,38,39].

The limitations of our study are as follows: 1) The GWAS data used in this study were derived from the Sardinian population. Although the Sardinian population has been extensively used in genetic analysis, some immune traits and associations might be driven by genetic variants more common in this specific region. To further validate these results, GWAS data on peripheral blood immune phenotypes from other ethnic groups are needed. Despite the geographical specificity of the population, the GWAS study analyzed 3,757 individuals and approximately 22 million genetic variants, exceeding the typical scope of small-scale studies. Moreover, advanced statistical methods were employed, such as linear mixed models adjusted for genomic relationship matrices, to mitigate potential biases caused by genetic drift, thus ensuring the validity of the GWAS. 2) The sample size of GWAS data for exposure and the number of SNPs obtained was comparatively small. Future studies require larger GWAS databases for immune cell traits. However, in our study, the F-statistic value was used as the criterion to measure the strength of IVs, and only IVs with F > 10 were used in subsequent analyses, making our findings reliable. 3) As the study was based on a European database, the conclusions may not apply to other ethnic groups, limiting the generalizability of our results. Therefore, further validation is needed using GWAS data from other ethnic groups [33,38,39].

## Conclusions

In summary, our study enhances our understanding of the role of the peripheral immune system in PD pathogenesis and demonstrates a causal relationship between monocyte surface *CX3CR1* expression and reduced PD risk. Additional functional studies are still needed to elucidate the mechanisms underlying these immune features associated with PD.

## Supporting information

S1 Fig Leave one out of sensitivity tests for *CX3CR1* on monocytes. Calculate the MR results of the remaining IVs after removing the IVs one by one.
(TIF)

S2 Fig Leave one out of sensitivity tests for *CX3CR1* on CD14+CD16+ monocytes. Calculate the MR results of the remaining IVs after removing the IVs one by one.
(TIF)

S1 Table Characterization of immunophenotypes. (XLSX)

S2 Table Summary of instrumental variables. (XLSX)

S3 Table Summary of the causal relationships of immune cell traits on Parkinson’s disease (PD) with various Mendelian randomization (MR) methods. (XLSX)

S4 Table Results of inverse variance weighted analysis post-FDR correction. (XLSX)

S5 Table Results of weighted median analysis post-FDR correction. (XLSX)

S6 Table Results of MR Egger analysis post-FDR correction. (XLSX)

S1 File Code available. (PDF)
