# Supplementary figures and images for: Peripheral immune cell traits and Parkinson’s disease: A Mendelian randomization study
Source: PLoS One. 2024 Mar 5;19(3):e0299026. doi: 10.1371/journal.pone.0299026 (PMC10914262; doi:10.1371/journal.pone.0299026)

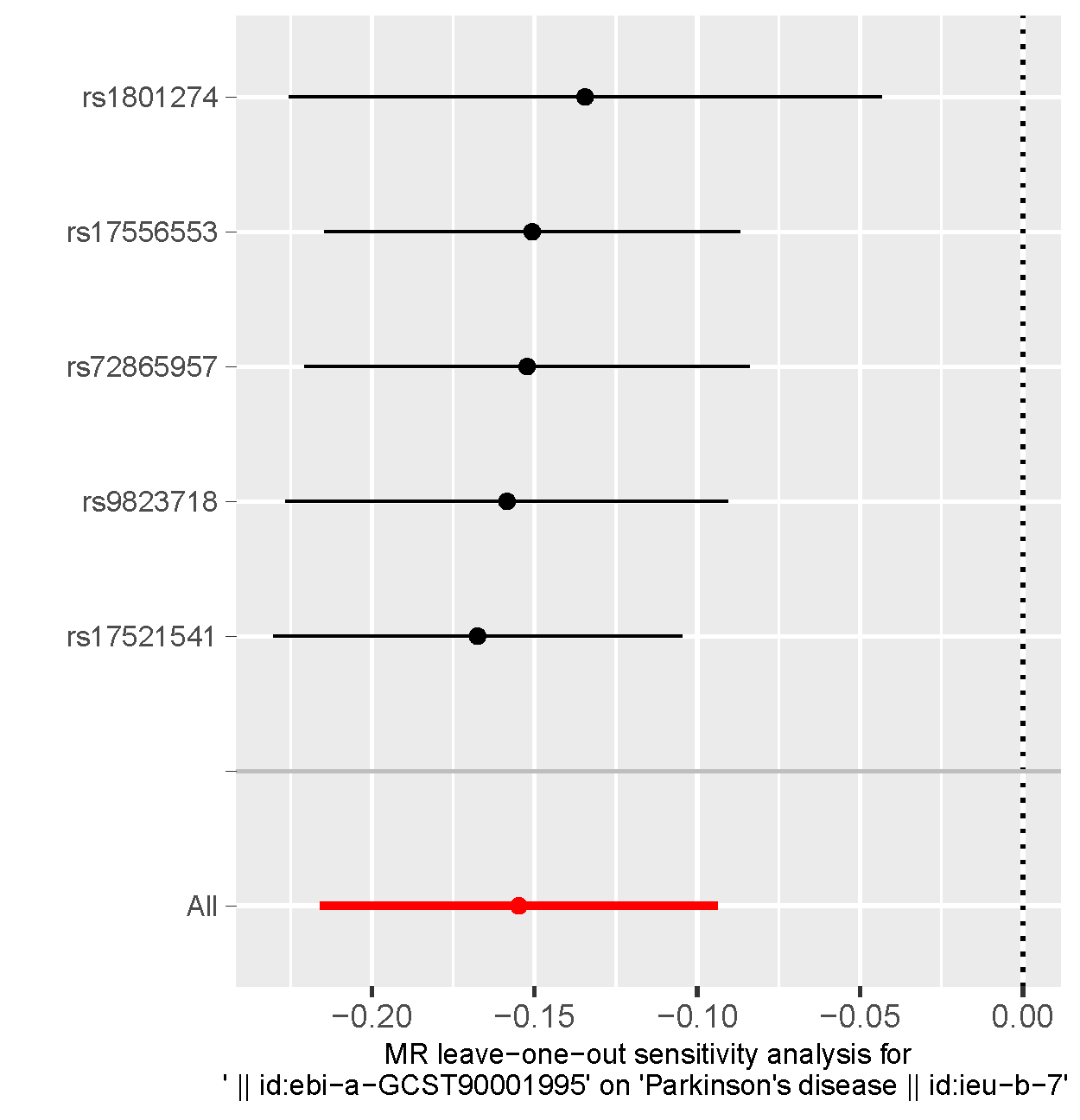

Supplement: S1 Fig — Calculate the MR results of the remaining IVs after removing the IVs one by one. (TIF) [file pone.0299026.s001.tif]

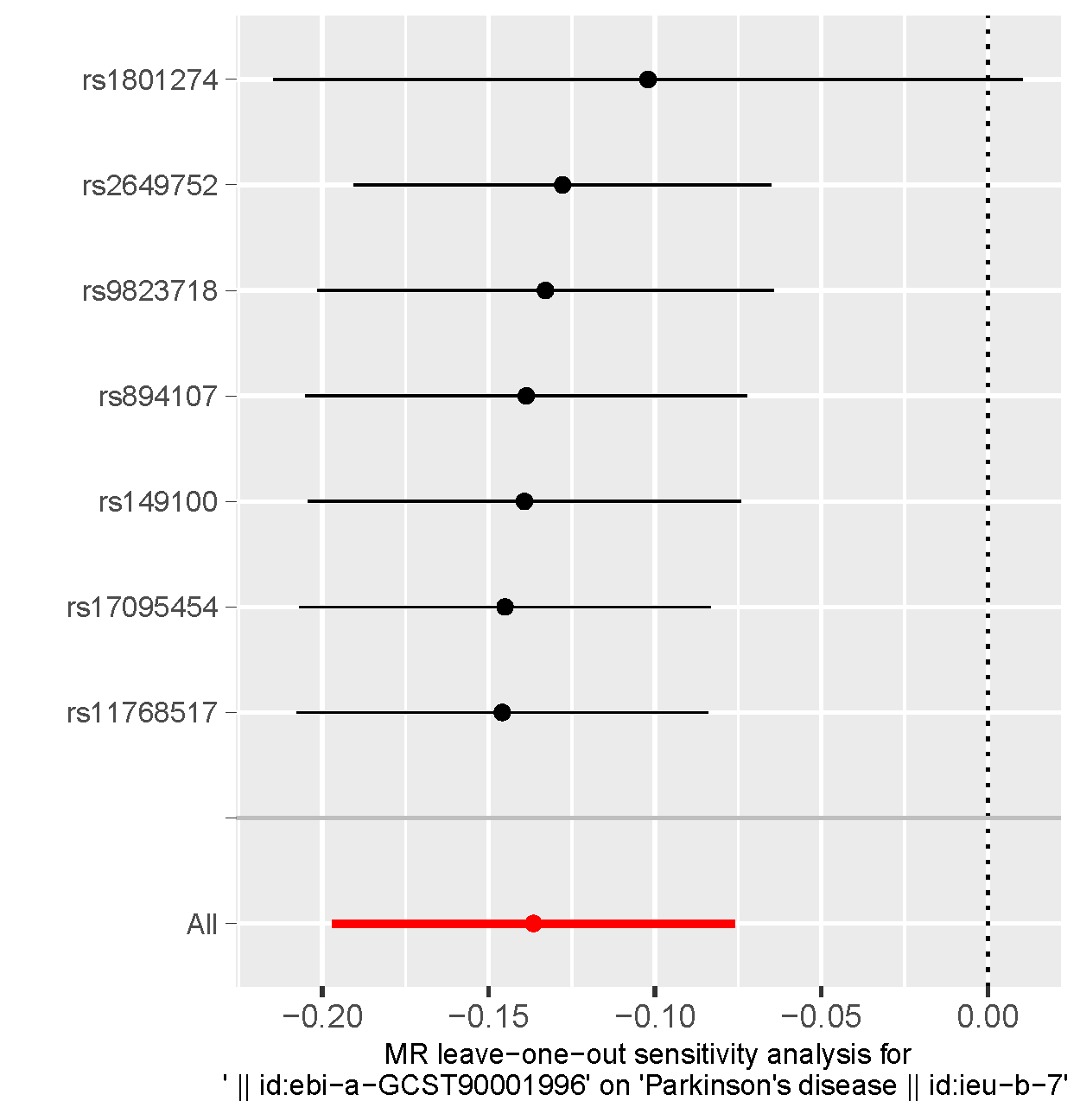

Supplement: S2 Fig — Calculate the MR results of the remaining IVs after removing the IVs one by one. (TIF) [file pone.0299026.s002.tif]
