# Supplementary material for: Peripheral immune cell traits and Parkinson’s disease: A Mendelian randomization study
Source: PLoS One. 2024 Mar 5;19(3):e0299026. doi: 10.1371/journal.pone.0299026 (PMC10914262; doi:10.1371/journal.pone.0299026)
Supplement: S1 File — (PDF) [file pone.0299026.s009.pdf]

```
library(TwoSampleMR)
library(MRPRESSO)
library(ggplot2)
library(ieugwasr)
datasets <- c(
'ebi-a-GCST90001391', 'ebi-a-GCST90001392', 'ebi-a-GCST90001393', 'ebi-a-
GCST90001394', 'ebi-a-GCST90001395', 'ebi-a-GCST90001396', 'ebi-a-GCST90001397',
'ebi-a-GCST90001398', 'ebi-a-GCST90001399', 'ebi-a-GCST90001400', 'ebi-a-
GCST90001401', 'ebi-a-GCST90001402', 'ebi-a-GCST90001403', 'ebi-a-GCST90001404',
'ebi-a-GCST90001405', 'ebi-a-GCST90001406', 'ebi-a-GCST90001407', 'ebi-a-
GCST90001408', 'ebi-a-GCST90001409', 'ebi-a-GCST90001410', 'ebi-a-GCST90001411',
'ebi-a-GCST90001412', 'ebi-a-GCST90001413', 'ebi-a-GCST90001414', 'ebi-a-
GCST90001415', 'ebi-a-GCST90001416', 'ebi-a-GCST90001417', 'ebi-a-GCST90001418',
'ebi-a-GCST90001419', 'ebi-a-GCST90001420', 'ebi-a-GCST90001421', 'ebi-a-
GCST90001422', 'ebi-a-GCST90001423', 'ebi-a-GCST90001424', 'ebi-a-GCST90001425',
'ebi-a-GCST90001426', 'ebi-a-GCST90001427', 'ebi-a-GCST90001428', 'ebi-a-
GCST90001429', 'ebi-a-GCST90001430', 'ebi-a-GCST90001431', 'ebi-a-GCST90001432',
'ebi-a-GCST90001433', 'ebi-a-GCST90001434', 'ebi-a-GCST90001435', 'ebi-a-
GCST90001436', 'ebi-a-GCST90001437', 'ebi-a-GCST90001438', 'ebi-a-GCST90001439',
'ebi-a-GCST90001440', 'ebi-a-GCST90001441', 'ebi-a-GCST90001442', 'ebi-a-
GCST90001443', 'ebi-a-GCST90001444', 'ebi-a-GCST90001445', 'ebi-a-GCST90001446',
'ebi-a-GCST90001447', 'ebi-a-GCST90001448', 'ebi-a-GCST90001449', 'ebi-a-
GCST90001450', 'ebi-a-GCST90001451', 'ebi-a-GCST90001452', 'ebi-a-GCST90001453',
'ebi-a-GCST90001454', 'ebi-a-GCST90001455', 'ebi-a-GCST90001456', 'ebi-a-
GCST90001457', 'ebi-a-GCST90001458', 'ebi-a-GCST90001459', 'ebi-a-GCST90001460',
'ebi-a-GCST90001461', 'ebi-a-GCST90001462', 'ebi-a-GCST90001463', 'ebi-a-
GCST90001464', 'ebi-a-GCST90001465', 'ebi-a-GCST90001466', 'ebi-a-GCST90001467',
'ebi-a-GCST90001468', 'ebi-a-GCST90001469', 'ebi-a-GCST90001470', 'ebi-a-
GCST90001471', 'ebi-a-GCST90001472', 'ebi-a-GCST90001473', 'ebi-a-GCST90001474',
'ebi-a-GCST90001475', 'ebi-a-GCST90001476', 'ebi-a-GCST90001477', 'ebi-a-
GCST90001478', 'ebi-a-GCST90001479', 'ebi-a-GCST90001480', 'ebi-a-GCST90001481',
'ebi-a-GCST90001482', 'ebi-a-GCST90001483', 'ebi-a-GCST90001484', 'ebi-a-
GCST90001485', 'ebi-a-GCST90001486', 'ebi-a-GCST90001487', 'ebi-a-GCST90001488',
'ebi-a-GCST90001489', 'ebi-a-GCST90001490', 'ebi-a-GCST90001491', 'ebi-a-
GCST90001492', 'ebi-a-GCST90001493', 'ebi-a-GCST90001494', 'ebi-a-GCST90001495',
'ebi-a-GCST90001496', 'ebi-a-GCST90001497', 'ebi-a-GCST90001498', 'ebi-a-
GCST90001499', 'ebi-a-GCST90001500', 'ebi-a-GCST90001501', 'ebi-a-GCST90001502',
'ebi-a-GCST90001503', 'ebi-a-GCST90001504', 'ebi-a-GCST90001505', 'ebi-a-
GCST90001506', 'ebi-a-GCST90001507', 'ebi-a-GCST90001508', 'ebi-a-GCST90001509',
'ebi-a-GCST90001510', 'ebi-a-GCST90001511', 'ebi-a-GCST90001512', 'ebi-a-
GCST90001513', 'ebi-a-GCST90001514', 'ebi-a-GCST90001515', 'ebi-a-GCST90001516',
'ebi-a-GCST90001517', 'ebi-a-GCST90001518', 'ebi-a-GCST90001519', 'ebi-a-
GCST90001520', 'ebi-a-GCST90001521', 'ebi-a-GCST90001522', 'ebi-a-GCST90001523',
'ebi-a-GCST90001524', 'ebi-a-GCST90001525', 'ebi-a-GCST90001526', 'ebi-a-
```

GCST90001527', 'ebi-a-GCST90001528', 'ebi-a-GCST90001529', 'ebi-a-GCST90001530', 'ebi-a-GCST90001531', 'ebi-a-GCST90001532', 'ebi-a-GCST90001533', 'ebi-a-GCST90001534', 'ebi-a-GCST90001535', 'ebi-a-GCST90001536', 'ebi-a-GCST90001537', 'ebi-a-GCST90001538', 'ebi-a-GCST90001539', 'ebi-a-GCST90001540', 'ebi-a-GCST90001541', 'ebi-a-GCST90001542', 'ebi-a-GCST90001543', 'ebi-a-GCST90001544', 'ebi-a-GCST90001545', 'ebi-a-GCST90001546', 'ebi-a-GCST90001547', 'ebi-a-GCST90001548', 'ebi-a-GCST90001549', 'ebi-a-GCST90001550', 'ebi-a-GCST90001551', 'ebi-a-GCST90001552', 'ebi-a-GCST90001553', 'ebi-a-GCST90001554', 'ebi-a-GCST90001555', 'ebi-a-GCST90001556', 'ebi-a-GCST90001557', 'ebi-a-GCST90001558', 'ebi-a-GCST90001559', 'ebi-a-GCST90001560', 'ebi-a-GCST90001561', 'ebi-a-GCST90001562', 'ebi-a-GCST90001563', 'ebi-a-GCST90001564', 'ebi-a-GCST90001565', 'ebi-a-GCST90001566', 'ebi-a-GCST90001567', 'ebi-a-GCST90001568', 'ebi-a-GCST90001569', 'ebi-a-GCST90001570', 'ebi-a-GCST90001571', 'ebi-a-GCST90001572', 'ebi-a-GCST90001573', 'ebi-a-GCST90001574', 'ebi-a-GCST90001575', 'ebi-a-GCST90001576', 'ebi-a-GCST90001577', 'ebi-a-GCST90001578', 'ebi-a-GCST90001579', 'ebi-a-GCST90001580', 'ebi-a-GCST90001581', 'ebi-a-GCST90001582', 'ebi-a-GCST90001583', 'ebi-a-GCST90001584', 'ebi-a-GCST90001585', 'ebi-a-GCST90001586', 'ebi-a-GCST90001587', 'ebi-a-GCST90001588', 'ebi-a-GCST90001589', 'ebi-a-GCST90001590', 'ebi-a-GCST90001591', 'ebi-a-GCST90001592', 'ebi-a-GCST90001593', 'ebi-a-GCST90001594', 'ebi-a-GCST90001595', 'ebi-a-GCST90001596', 'ebi-a-GCST90001597', 'ebi-a-GCST90001598', 'ebi-a-GCST90001599', 'ebi-a-GCST90001600', 'ebi-a-GCST90001601', 'ebi-a-GCST90001602', 'ebi-a-GCST90001603', 'ebi-a-GCST90001604', 'ebi-a-GCST90001605', 'ebi-a-GCST90001606', 'ebi-a-GCST90001607', 'ebi-a-GCST90001608', 'ebi-a-GCST90001609', 'ebi-a-GCST90001610', 'ebi-a-GCST90001611', 'ebi-a-GCST90001612', 'ebi-a-GCST90001613', 'ebi-a-GCST90001614', 'ebi-a-GCST90001615', 'ebi-a-GCST90001616', 'ebi-a-GCST90001617', 'ebi-a-GCST90001618', 'ebi-a-GCST90001619', 'ebi-a-GCST90001620', 'ebi-a-GCST90001621', 'ebi-a-GCST90001622', 'ebi-a-GCST90001623', 'ebi-a-GCST90001624', 'ebi-a-GCST90001625', 'ebi-a-GCST90001626', 'ebi-a-GCST90001627', 'ebi-a-GCST90001628', 'ebi-a-GCST90001629', 'ebi-a-GCST90001630', 'ebi-a-GCST90001631', 'ebi-a-GCST90001632', 'ebi-a-GCST90001633', 'ebi-a-GCST90001634', 'ebi-a-GCST90001635', 'ebi-a-GCST90001636', 'ebi-a-GCST90001637', 'ebi-a-GCST90001638', 'ebi-a-GCST90001639', 'ebi-a-GCST90001640', 'ebi-a-GCST90001641', 'ebi-a-GCST90001642', 'ebi-a-GCST90001643', 'ebi-a-GCST90001644', 'ebi-a-GCST90001645', 'ebi-a-GCST90001646', 'ebi-a-GCST90001647', 'ebi-a-GCST90001648', 'ebi-a-GCST90001649', 'ebi-a-GCST90001650', 'ebi-a-GCST90001651', 'ebi-a-GCST90001652', 'ebi-a-GCST90001653', 'ebi-a-GCST90001654', 'ebi-a-GCST90001655', 'ebi-a-GCST90001656', 'ebi-a-GCST90001657', 'ebi-a-GCST90001658', 'ebi-a-GCST90001659', 'ebi-a-GCST90001660', 'ebi-a-GCST90001661', 'ebi-a-GCST90001662', 'ebi-a-GCST90001663', 'ebi-a-GCST90001664', 'ebi-a-GCST90001665', 'ebi-a-GCST90001666', 'ebi-a-GCST90001667', 'ebi-a-GCST90001668', 'ebi-a-GCST90001669', 'ebi-a-GCST90001670', 'ebi-a-GCST90001671', 'ebi-a-GCST90001672', 'ebi-a-GCST90001673', 'ebi-a-GCST90001674', 'ebi-a-GCST90001675', 'ebi-a-GCST90001676', 'ebi-a-GCST90001677', 'ebi-a-GCST90001678', 'ebi-a-GCST90001679', 'ebi-a-GCST90001680', 'ebi-a-

GCST90001681', 'ebi-a-GCST90001682', 'ebi-a-GCST90001683', 'ebi-a-GCST90001684', 'ebi-a-GCST90001685', 'ebi-a-GCST90001686', 'ebi-a-GCST90001687', 'ebi-a-GCST90001688', 'ebi-a-GCST90001689', 'ebi-a-GCST90001690', 'ebi-a-GCST90001691', 'ebi-a-GCST90001692', 'ebi-a-GCST90001693', 'ebi-a-GCST90001694', 'ebi-a-GCST90001695', 'ebi-a-GCST90001696', 'ebi-a-GCST90001697', 'ebi-a-GCST90001698', 'ebi-a-GCST90001699', 'ebi-a-GCST90001700', 'ebi-a-GCST90001701', 'ebi-a-GCST90001702', 'ebi-a-GCST90001703', 'ebi-a-GCST90001704', 'ebi-a-GCST90001705', 'ebi-a-GCST90001706', 'ebi-a-GCST90001707', 'ebi-a-GCST90001708', 'ebi-a-GCST90001709', 'ebi-a-GCST90001710', 'ebi-a-GCST90001711', 'ebi-a-GCST90001712', 'ebi-a-GCST90001713', 'ebi-a-GCST90001714', 'ebi-a-GCST90001715', 'ebi-a-GCST90001716', 'ebi-a-GCST90001717', 'ebi-a-GCST90001718', 'ebi-a-GCST90001719', 'ebi-a-GCST90001720', 'ebi-a-GCST90001721', 'ebi-a-GCST90001722', 'ebi-a-GCST90001723', 'ebi-a-GCST90001724', 'ebi-a-GCST90001725', 'ebi-a-GCST90001726', 'ebi-a-GCST90001727', 'ebi-a-GCST90001728', 'ebi-a-GCST90001729', 'ebi-a-GCST90001730', 'ebi-a-GCST90001731', 'ebi-a-GCST90001732', 'ebi-a-GCST90001733', 'ebi-a-GCST90001734', 'ebi-a-GCST90001735', 'ebi-a-GCST90001736', 'ebi-a-GCST90001737', 'ebi-a-GCST90001738', 'ebi-a-GCST90001739', 'ebi-a-GCST90001740', 'ebi-a-GCST90001741', 'ebi-a-GCST90001742', 'ebi-a-GCST90001743', 'ebi-a-GCST90001744', 'ebi-a-GCST90001745', 'ebi-a-GCST90001746', 'ebi-a-GCST90001747', 'ebi-a-GCST90001748', 'ebi-a-GCST90001749', 'ebi-a-GCST90001750', 'ebi-a-GCST90001751', 'ebi-a-GCST90001752', 'ebi-a-GCST90001753', 'ebi-a-GCST90001754', 'ebi-a-GCST90001755', 'ebi-a-GCST90001756', 'ebi-a-GCST90001757', 'ebi-a-GCST90001758', 'ebi-a-GCST90001759', 'ebi-a-GCST90001760', 'ebi-a-GCST90001761', 'ebi-a-GCST90001762', 'ebi-a-GCST90001763', 'ebi-a-GCST90001764', 'ebi-a-GCST90001765', 'ebi-a-GCST90001766', 'ebi-a-GCST90001767', 'ebi-a-GCST90001768', 'ebi-a-GCST90001769', 'ebi-a-GCST90001770', 'ebi-a-GCST90001771', 'ebi-a-GCST90001772', 'ebi-a-GCST90001773', 'ebi-a-GCST90001774', 'ebi-a-GCST90001775', 'ebi-a-GCST90001776', 'ebi-a-GCST90001777', 'ebi-a-GCST90001778', 'ebi-a-GCST90001779', 'ebi-a-GCST90001780', 'ebi-a-GCST90001781', 'ebi-a-GCST90001782', 'ebi-a-GCST90001783', 'ebi-a-GCST90001784', 'ebi-a-GCST90001785', 'ebi-a-GCST90001786', 'ebi-a-GCST90001787', 'ebi-a-GCST90001788', 'ebi-a-GCST90001789', 'ebi-a-GCST90001790', 'ebi-a-GCST90001791', 'ebi-a-GCST90001792', 'ebi-a-GCST90001793', 'ebi-a-GCST90001794', 'ebi-a-GCST90001795', 'ebi-a-GCST90001796', 'ebi-a-GCST90001797', 'ebi-a-GCST90001798', 'ebi-a-GCST90001799', 'ebi-a-GCST90001800', 'ebi-a-GCST90001801', 'ebi-a-GCST90001802', 'ebi-a-GCST90001803', 'ebi-a-GCST90001804', 'ebi-a-GCST90001805', 'ebi-a-GCST90001806', 'ebi-a-GCST90001807', 'ebi-a-GCST90001808', 'ebi-a-GCST90001809', 'ebi-a-GCST90001810', 'ebi-a-GCST90001811', 'ebi-a-GCST90001812', 'ebi-a-GCST90001813', 'ebi-a-GCST90001814', 'ebi-a-GCST90001815', 'ebi-a-GCST90001816', 'ebi-a-GCST90001817', 'ebi-a-GCST90001818', 'ebi-a-GCST90001819', 'ebi-a-GCST90001820', 'ebi-a-GCST90001821', 'ebi-a-GCST90001822', 'ebi-a-GCST90001823', 'ebi-a-GCST90001824', 'ebi-a-GCST90001825', 'ebi-a-GCST90001826', 'ebi-a-GCST90001827', 'ebi-a-GCST90001828', 'ebi-a-GCST90001829', 'ebi-a-GCST90001830', 'ebi-a-GCST90001831', 'ebi-a-GCST90001832', 'ebi-a-GCST90001833', 'ebi-a-GCST90001834', 'ebi-a-

GCST90001835', 'ebi-a-GCST90001836', 'ebi-a-GCST90001837', 'ebi-a-GCST90001838', 'ebi-a-GCST90001839', 'ebi-a-GCST90001840', 'ebi-a-GCST90001841', 'ebi-a-GCST90001842', 'ebi-a-GCST90001843', 'ebi-a-GCST90001844', 'ebi-a-GCST90001845', 'ebi-a-GCST90001846', 'ebi-a-GCST90001847', 'ebi-a-GCST90001848', 'ebi-a-GCST90001849', 'ebi-a-GCST90001850', 'ebi-a-GCST90001851', 'ebi-a-GCST90001852', 'ebi-a-GCST90001853', 'ebi-a-GCST90001854', 'ebi-a-GCST90001855', 'ebi-a-GCST90001856', 'ebi-a-GCST90001857', 'ebi-a-GCST90001858', 'ebi-a-GCST90001859', 'ebi-a-GCST90001860', 'ebi-a-GCST90001861', 'ebi-a-GCST90001862', 'ebi-a-GCST90001863', 'ebi-a-GCST90001864', 'ebi-a-GCST90001865', 'ebi-a-GCST90001866', 'ebi-a-GCST90001867', 'ebi-a-GCST90001868', 'ebi-a-GCST90001869', 'ebi-a-GCST90001870', 'ebi-a-GCST90001871', 'ebi-a-GCST90001872', 'ebi-a-GCST90001873', 'ebi-a-GCST90001874', 'ebi-a-GCST90001875', 'ebi-a-GCST90001876', 'ebi-a-GCST90001877', 'ebi-a-GCST90001878', 'ebi-a-GCST90001879', 'ebi-a-GCST90001880', 'ebi-a-GCST90001881', 'ebi-a-GCST90001882', 'ebi-a-GCST90001883', 'ebi-a-GCST90001884', 'ebi-a-GCST90001885', 'ebi-a-GCST90001886', 'ebi-a-GCST90001887', 'ebi-a-GCST90001888', 'ebi-a-GCST90001889', 'ebi-a-GCST90001890', 'ebi-a-GCST90001891', 'ebi-a-GCST90001892', 'ebi-a-GCST90001893', 'ebi-a-GCST90001894', 'ebi-a-GCST90001895', 'ebi-a-GCST90001896', 'ebi-a-GCST90001897', 'ebi-a-GCST90001898', 'ebi-a-GCST90001899', 'ebi-a-GCST90001900', 'ebi-a-GCST90001901', 'ebi-a-GCST90001902', 'ebi-a-GCST90001903', 'ebi-a-GCST90001904', 'ebi-a-GCST90001905', 'ebi-a-GCST90001906', 'ebi-a-GCST90001907', 'ebi-a-GCST90001908', 'ebi-a-GCST90001909', 'ebi-a-GCST90001910', 'ebi-a-GCST90001911', 'ebi-a-GCST90001912', 'ebi-a-GCST90001913', 'ebi-a-GCST90001914', 'ebi-a-GCST90001915', 'ebi-a-GCST90001916', 'ebi-a-GCST90001917', 'ebi-a-GCST90001918', 'ebi-a-GCST90001919', 'ebi-a-GCST90001920', 'ebi-a-GCST90001921', 'ebi-a-GCST90001922', 'ebi-a-GCST90001923', 'ebi-a-GCST90001924', 'ebi-a-GCST90001925', 'ebi-a-GCST90001926', 'ebi-a-GCST90001927', 'ebi-a-GCST90001928', 'ebi-a-GCST90001929', 'ebi-a-GCST90001930', 'ebi-a-GCST90001931', 'ebi-a-GCST90001932', 'ebi-a-GCST90001933', 'ebi-a-GCST90001934', 'ebi-a-GCST90001935', 'ebi-a-GCST90001936', 'ebi-a-GCST90001937', 'ebi-a-GCST90001938', 'ebi-a-GCST90001939', 'ebi-a-GCST90001940', 'ebi-a-GCST90001941', 'ebi-a-GCST90001942', 'ebi-a-GCST90001943', 'ebi-a-GCST90001944', 'ebi-a-GCST90001945', 'ebi-a-GCST90001946', 'ebi-a-GCST90001947', 'ebi-a-GCST90001948', 'ebi-a-GCST90001949', 'ebi-a-GCST90001950', 'ebi-a-GCST90001951', 'ebi-a-GCST90001952', 'ebi-a-GCST90001953', 'ebi-a-GCST90001954', 'ebi-a-GCST90001955', 'ebi-a-GCST90001956', 'ebi-a-GCST90001957', 'ebi-a-GCST90001958', 'ebi-a-GCST90001959', 'ebi-a-GCST90001960', 'ebi-a-GCST90001961', 'ebi-a-GCST90001962', 'ebi-a-GCST90001963', 'ebi-a-GCST90001964', 'ebi-a-GCST90001965', 'ebi-a-GCST90001966', 'ebi-a-GCST90001967', 'ebi-a-GCST90001968', 'ebi-a-GCST90001969', 'ebi-a-GCST90001970', 'ebi-a-GCST90001971', 'ebi-a-GCST90001972', 'ebi-a-GCST90001973', 'ebi-a-GCST90001974', 'ebi-a-GCST90001975', 'ebi-a-GCST90001976', 'ebi-a-GCST90001977', 'ebi-a-GCST90001978', 'ebi-a-GCST90001979', 'ebi-a-GCST90001980', 'ebi-a-GCST90001981', 'ebi-a-GCST90001982', 'ebi-a-GCST90001983', 'ebi-a-GCST90001984', 'ebi-a-GCST90001985', 'ebi-a-GCST90001986', 'ebi-a-GCST90001987', 'ebi-a-GCST90001988', 'ebi-a-

```
GCST90001989', 'ebi-a-GCST90001990', 'ebi-a-GCST90001991', 'ebi-a-GCST90001992',
'ebi-a-GCST90001993', 'ebi-a-GCST90001994', 'ebi-a-GCST90001995', 'ebi-a-
GCST90001996', 'ebi-a-GCST90001997', 'ebi-a-GCST90001998', 'ebi-a-GCST90001999',
'ebi-a-GCST90002000', 'ebi-a-GCST90002001', 'ebi-a-GCST90002002', 'ebi-a-
GCST90002003', 'ebi-a-GCST90002004', 'ebi-a-GCST90002005', 'ebi-a-GCST90002006',
'ebi-a-GCST90002007', 'ebi-a-GCST90002008', 'ebi-a-GCST90002009', 'ebi-a-
GCST90002010', 'ebi-a-GCST90002011', 'ebi-a-GCST90002012', 'ebi-a-GCST90002013',
'ebi-a-GCST90002014', 'ebi-a-GCST90002015', 'ebi-a-GCST90002016', 'ebi-a-
GCST90002017', 'ebi-a-GCST90002018', 'ebi-a-GCST90002019', 'ebi-a-GCST90002020',
'ebi-a-GCST90002021', 'ebi-a-GCST90002022', 'ebi-a-GCST90002023', 'ebi-a-
GCST90002024', 'ebi-a-GCST90002025', 'ebi-a-GCST90002026', 'ebi-a-GCST90002027',
'ebi-a-GCST90002028', 'ebi-a-GCST90002029', 'ebi-a-GCST90002030', 'ebi-a-
GCST90002031', 'ebi-a-GCST90002032', 'ebi-a-GCST90002033', 'ebi-a-GCST90002034',
'ebi-a-GCST90002035', 'ebi-a-GCST90002036', 'ebi-a-GCST90002037', 'ebi-a-
GCST90002038', 'ebi-a-GCST90002039', 'ebi-a-GCST90002040', 'ebi-a-GCST90002041',
'ebi-a-GCST90002042', 'ebi-a-GCST90002043', 'ebi-a-GCST90002044', 'ebi-a-
GCST90002045', 'ebi-a-GCST90002046', 'ebi-a-GCST90002047', 'ebi-a-GCST90002048',
'ebi-a-GCST90002049', 'ebi-a-GCST90002050', 'ebi-a-GCST90002051', 'ebi-a-
GCST90002052', 'ebi-a-GCST90002053', 'ebi-a-GCST90002054', 'ebi-a-GCST90002055',
'ebi-a-GCST90002056', 'ebi-a-GCST90002057', 'ebi-a-GCST90002058', 'ebi-a-
GCST90002059', 'ebi-a-GCST90002060', 'ebi-a-GCST90002061', 'ebi-a-GCST90002062',
'ebi-a-GCST90002063', 'ebi-a-GCST90002064', 'ebi-a-GCST90002065', 'ebi-a-
GCST90002066', 'ebi-a-GCST90002067', 'ebi-a-GCST90002068', 'ebi-a-GCST90002069',
'ebi-a-GCST90002070', 'ebi-a-GCST90002071', 'ebi-a-GCST90002072', 'ebi-a-
GCST90002073', 'ebi-a-GCST90002074', 'ebi-a-GCST90002075', 'ebi-a-GCST90002076',
'ebi-a-GCST90002077', 'ebi-a-GCST90002078', 'ebi-a-GCST90002079', 'ebi-a-
GCST90002080', 'ebi-a-GCST90002081', 'ebi-a-GCST90002082', 'ebi-a-GCST90002083',
'ebi-a-GCST90002084', 'ebi-a-GCST90002085', 'ebi-a-GCST90002086', 'ebi-a-
GCST90002087', 'ebi-a-GCST90002088', 'ebi-a-GCST90002089', 'ebi-a-GCST90002090',
'ebi-a-GCST90002091', 'ebi-a-GCST90002092', 'ebi-a-GCST90002093', 'ebi-a-
GCST90002094', 'ebi-a-GCST90002095', 'ebi-a-GCST90002096', 'ebi-a-GCST90002097',
'ebi-a-GCST90002098', 'ebi-a-GCST90002099', 'ebi-a-GCST90002100', 'ebi-a-
GCST90002101', 'ebi-a-GCST90002102', 'ebi-a-GCST90002103', 'ebi-a-GCST90002104',
'ebi-a-GCST90002105', 'ebi-a-GCST90002106', 'ebi-a-GCST90002107', 'ebi-a-
GCST90002108', 'ebi-a-GCST90002109', 'ebi-a-GCST90002110', 'ebi-a-GCST90002111',
'ebi-a-GCST90002112', 'ebi-a-GCST90002113', 'ebi-a-GCST90002114', 'ebi-a-
GCST90002115', 'ebi-a-GCST90002116', 'ebi-a-GCST90002117', 'ebi-a-GCST90002118',
'ebi-a-GCST90002119', 'ebi-a-GCST90002120', 'ebi-a-GCST90002121') #Fill in all IDs to
run the loop
error_log <- file("error_log.txt", open = "w")

# Loop through the data set
for (dataset in datasets) {
  cat(paste("Processing dataset: ", dataset, "\n"))
```

```
tryCatch({
    ebi_a <- extract_instruments(
      outcomes = dataset,
      clump = TRUE, r2 = 0.001, p1 = 1e-6,
      kb = 10000, access_token = NULL
    )
    ebi_a<-subset(X1,SNP %in% X11$rsid)
    ebi_a$R2 <- 2 * (1 - ebi_a$eaf.exposure) * ebi_a$eaf.exposure *
(ebi_a$beta.exposure)^2
    ebi_a$F <- (ebi_a$R2) / (1 - ebi_a$R2) * (ebi_a$samplesize.exposure - 2)

    # Ensure that F is greater than 10
    ebi_a <- subset(ebi_a, F > 10)

    write.csv(ebi_a, file = paste0(dataset, "_R2_F.csv"))

    pd_out <- extract_outcome_data(
      snps = ebi_a$SNP,
      outcomes = 'ieu-b-7',
      proxies = FALSE,
      maf_threshold = 0.01,
      access_token = NULL
    )

    mydata <- harmonise_data(
      exposure_dat = ebi_a,
      outcome_dat = pd_out,
      action = 2
    )

    res <- mr(mydata, method_list = c("mr_ivw", "mr_weighted_median",
"mr_egger_regression"))
    generate_odds_ratios(res)
    mr_res <- generate_odds_ratios(res)
    write.csv(mr_res, file = paste0(dataset, "_pd.csv"))

    # Heterogeneity test
    het <- mr_heterogeneity(mydata)
    print(het)
    write.csv(het, file = paste0(dataset, "_heterogeneity.csv"))
    pleio <- mr_pleiotropy_test(mydata)
    pleio
    write.csv(pleio, file = paste0(dataset, "_pleiotropy.csv"))
```

```
    # Execute mr_presso
    mr_presso <- capture.output({
      mr_presso(BetaOutcome = "beta.outcome", BetaExposure = "beta.exposure",
SdOutcome = "se.outcome", SdExposure = "se.exposure",
                OUTLIERtest = TRUE, DISTORTIONtest = TRUE, data = mydata,
NbDistribution = 1000,
                SignifThreshold = 0.05)
    })

    output_file <- paste0(dataset, "_mr_presso.txt")

    writeLines(mr_presso, con = output_file)

    p1 <- mr_scatter_plot(res, mydata)
    ggsave(p1[[1]], file = paste0(dataset, "_scatter_plot.pdf"), width = 7, height = 7)

    # Leave one out test
    single <- mr_leaveoneout(mydata)

    # Mapping the forest - single SNP test
    res_single <- mr_singlesnp(mydata)

    p2 <- mr_funnel_plot(res_single)
    ggsave(p2[[1]], file = paste0(dataset, "_funnel_plot.pdf"), width = 7, height = 7)

  }, error = function(e) {
    cat(paste("Error processing dataset: ", dataset, "\n"))
    cat(paste("Error message: ", conditionMessage(e), "\n"))
    cat("\n", file = error_log, append = TRUE)
  })
}

close(error_log)
```
